# Understanding the dynamics of terrorism events with multiple-discipline datasets and machine learning approach

**DOI:** 10.1371/journal.pone.0179057

**Published:** 2017-06-07

**Authors:** Fangyu Ding, Quansheng Ge, Dong Jiang, Jingying Fu, Mengmeng Hao

**Affiliations:** 1 Institute of Geographical Sciences and Natural Resources Research, Chinese Academy of Sciences, Beijing, China; 2 College of Resources and Environment, University of Chinese Academy of Sciences, Beijing, China; National Chiao Tung University College of Biological Science and Technology, TAIWAN

## Abstract

Terror events can cause profound consequences for the whole society. Finding out the regularity of terrorist attacks has important meaning for the global counter-terrorism strategy. In the present study, we demonstrate a novel method using relatively popular and robust machine learning methods to simulate the risk of terrorist attacks at a global scale based on multiple resources, long time series and globally distributed datasets. Historical data from 1970 to 2015 was adopted to train and evaluate machine learning models. The model performed fairly well in predicting the places where terror events might occur in 2015, with a success rate of 96.6%. Moreover, it is noteworthy that the model with optimized tuning parameter values successfully predicted 2,037 terrorism event locations where a terrorist attack had never happened before.

## Introduction

Terrorism is considered to be a major threat to society [1–3]. According to the Global Terrorism Database (GTD), more than 14800 terrorism events of different types occurred globally in 2015 alone[4], which not only caused the deaths of 38430 people but also caused the world to panic[5–7]. Great efforts have been made to seek explanations of various issues related to terrorist threats[8, 9]. However, the prediction of the occurrence of a certain event is still a difficult task of great complexity and uncertainty[10, 11]. Currently, data-driven models, which are direct learning patterns from large volumes of high-dimensional training data, have achieved some successes in a variety of fields[12–15], which provide a new perspective to solve this problem.

Previous studies have showed that natural[16] and social[17] factors are used in agent-based method to simulate the potential future scenarios of violent conflict, but the underlying mechanisms are still not well-understood. Everything is connected. Perhaps, there are some regularity of terrorist attacks[18]. Thus, the typical intuition of geographers posits the question of whether the occurrences of the terrorism events may be influenced or controlled by sets of geographical, natural and social issues (factors). If these patterns exist, then they can be discovered through machine learning approach.

Given the availability of global data, we collected three social elements, such as ethnic diversity, major drug regions and population density. Moreover, nighttime light are also included, which has been widely verified for monitoring economic activity[19]. Meantime, some natural elements were selected in the present study, including average precipitation, average temperature and topography. Considering the difference of these factors in space, latitude, longitude, distance to a major navigable lake, distance to an ice-free ocean, and distance to a major navigable river were used to express geospatial information. As a result, a total of 12 factors were quantified at a global scale (0.1 × 0.1 degree). Thanks to the excellent job of the STAR group at the University of Maryland, GTD was established, and more than 150,000 events were recorded in detail. We rasterized the location of terror events that occurred from 1970 to 2014, dividing units into 2 classes according to the frequency of a location and the consequence (i.e., the number of deaths).

**Fig 1:**
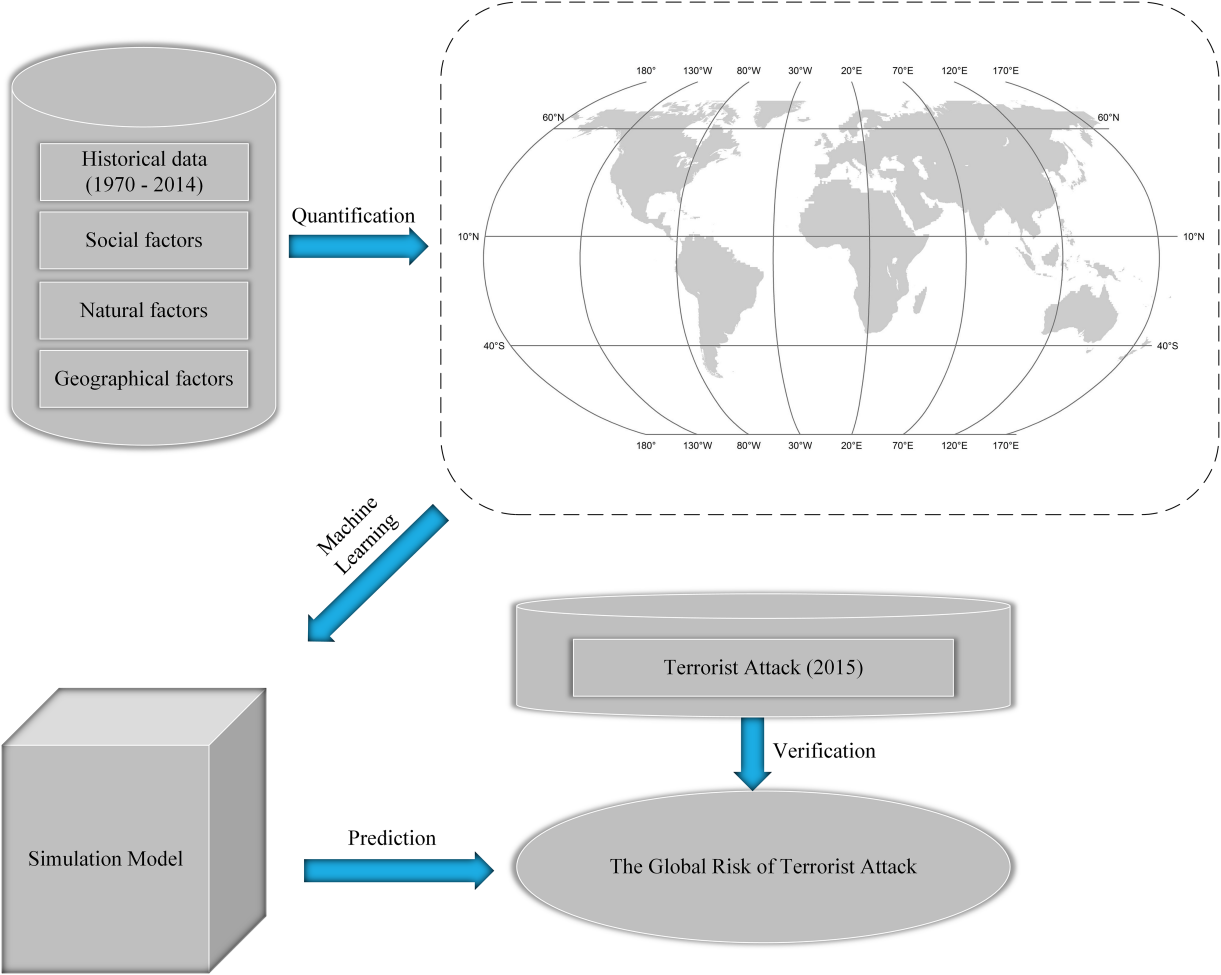
Workflow of the simulation the risk of terrorist attacks in all regions worldwide.

The specific flowchart is shown in Fig 1. To create simulation model, we develop a series of programs and use geographic Information Systems (GIS) software to preprocess various types of data and to select a sample dataset (see Methods). Three relatively popular machine learning algorithms are used in the present study: neural network (NNET), support vector machine (SVM), and random forest (RF). Additionally, the training approach and testing of the machine learning models are described in the Methods. Notably, the research scope is limited to the region between 75°N and 55°S.

## Methods

### Data preprocessing

The types and sources of data used in this research are shown in Table 1. GIS software and the C++ programming language were used to preprocess the datasets, including ArcMap 10.2 (http://www.esrichina.com.cn/), GDAL 2.1.0 (http://www.gdal.org/) and Proj.4 (https://github.com/OSGeo/proj.4). WGS-84 was selected as the geographic coordinate system for all of the data used in this research.

**Table 1:** Data used to obtain the factors of global terrorist attacks.

Data type	Source	Description	Publisher
Historical data	The Global Terrorism Database (GTD)	Table	National Consortium for the Study of Terrorism and Responses to Terrorism (START), University of Maryland, https://www.start.umd.edu/gtd/
Latitude			
Longitude			
Distance to major navigable lake	G-Econ 4.0	Table	Yale University, http://gecon.yale.edu/
Distance to major navigable river			
Distance to ice-free ocean			
Average precipitation			
Average temperature			
Ethnic diversity	GeoEPR, the Ethnic Power Relations dataset, version 2014	Polygon data	Center for Comparative and International Studies (CIS), International Conflict Research, ETH Zurich, http://www.icr.ethz.ch/data/index
Major drug regions	World drug report	Table	Division for Policy Analysis and Public Affairs, United Nations Office on Drugs and Crime, http://www.unvienna.org/unov/en/unodc.html
Nighttime lights	Nighttime Lights of the World, 2013	Grid	The Earth Observation Group, NOAA, http://ngdc.noaa.gov/eog/index.html
Population density	Population density of the World, 2000	Grid	NASA's Earth Observatory, http://neo.sci.gsfc.nasa.gov/
Topography	Digital elevation model (DEM), 2000		

Based on GTD, we can obtain the location of each incident and convert information on terrorist events around the world from 1970 to 2014 into raster data. Pixels of 0.1×0.1 degrees resolution were chosen as the units to statistically determine the number of terrorist events and the total number of fatalities. In the present study, an assessment unit is considered as having a high terrorist attack probability if there have been a terrorist attack causing casualties in the past; otherwise, it has a low probability. Class values of 1 and 0 were given for these types. In addition, we can obtain the latitude and longitude information of the centre point of each unit.

Based on G-Econ 4.0, a dataset on economic activity for the world, we can obtain the raster data of 5 factors, namely distance to major navigable lake (km), distance to major navigable river (km), distance to ice-free ocean (km), average precipitation (mm/year) and average temperature (°C) from 1980 to 2008. Then, we resample the above mentioned raster data into 0.1-degree longitude by 0.1-degree latitude resolution using ArcMap 10.2 because each terrestrial observation of G-Econ was measured at a 1-degree longitude by 1-degree latitude resolution at a global scale.

The ethnic distribution map was derived from GeoEPR at a 0.01-degree longitude by 0.01-degree latitude resolution using the ArcMap 10.2 “Polygon to Raster” function. In the present study, we assume that there are no intrinsic differences between ethnic groups with respect to terror events, but the conflict of different cultural beliefs with neighbouring regions may result in terror events. We used 0.1×0.1 degrees resolution pixels as the units to statistically determine the number of different ethnic groups in each region.

Major drug regions were derived from world drug report and national administrative boundaries (http://www.gadm.org/) using the ArcMap 10.2 “Polygon to Raster” and “Resample” functions. The remaining factors (nighttime lights, population density and topography) were calculated from their respective data sources and resampled to 0.1×0.1 degrees resolution pixels using ArcMap 10.2.

Another point to note is that the twelve potential factors mentioned above have different units. To facilitate the training step of models, they were normalized as the Eq 1 below:

$$P_{j}^{\prime }=\frac{P_{j}-P_{\text{min}}}{P_{\text{max}}-P_{\text{min}}}(j=1,2,3\ldots ,\text{n})$$ (1)

where $P_{j}^{\prime }$ is the normalized value between 0 and 1; *P*_*j*_ is the value of the factor; *P*_min_ is the minimum value; *P*_max_ is the maximum value; and n is the number of data.

### Sampling data

**Fig 2:**
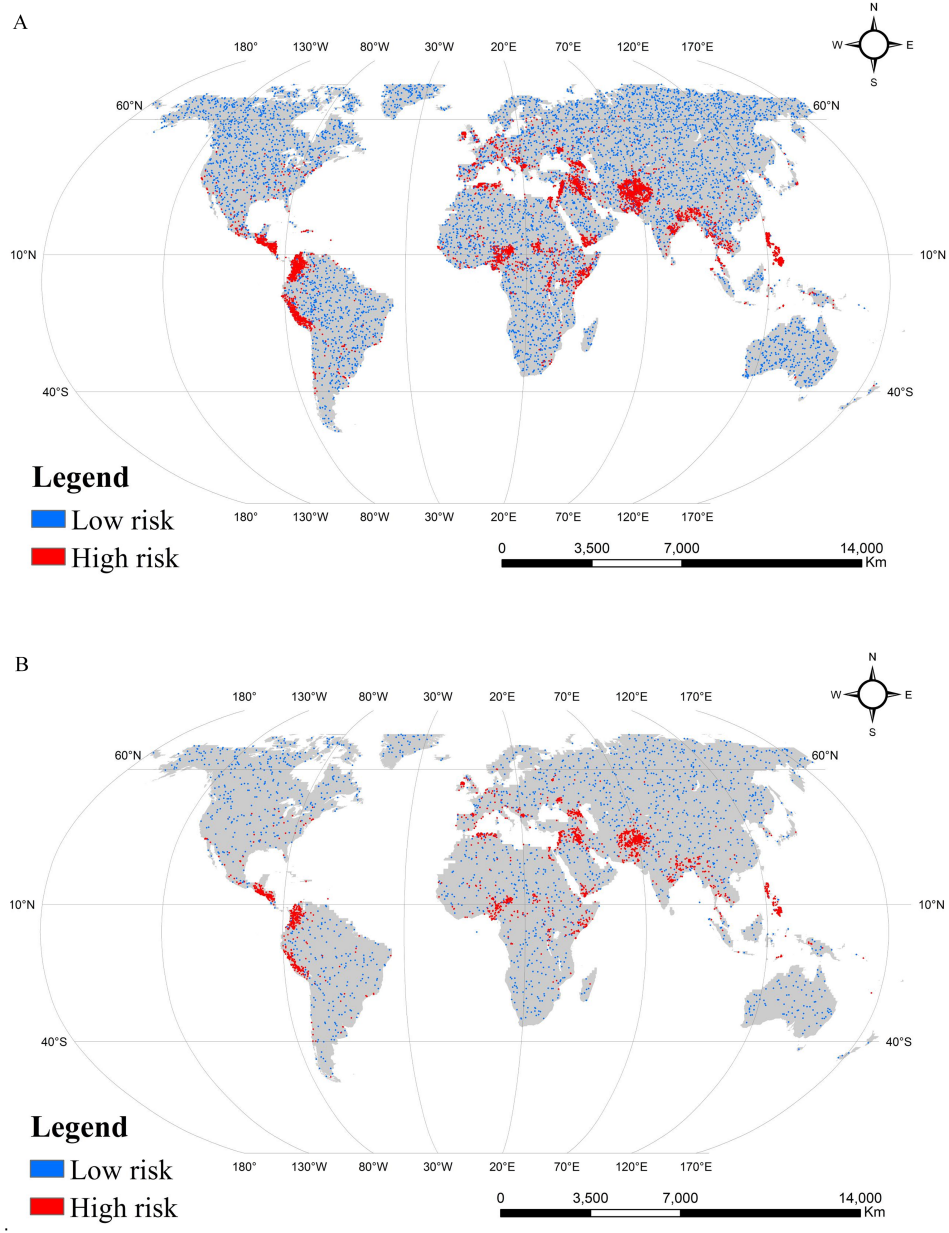
Spatial distribution of samples. (A) Training samples with 11,978 assessment units. Among the units, 5,989 have experienced terrorist attacks that resulted in casualties (= high risk), whereas 5,989 have not (= low risk). (B) Validation samples with 3,992 assessment units. Among the units, 1,996 have experienced terrorist attacks that resulted in casualties (= high risk), whereas 1,996 have not (= low risk).

The values of the attributes of a unit were gathered from the same location at each data layer and together constitute a sample row. If the location of some terrorist attacks is close, they will be recorded in the same statistical unit. Besides, small parts of units that were missing some factors’ information were excluded. In the present research, pixels where terrorist attacks resulted in more than 0 of the number of casualties were selected, and the same number of pixels were randomly selected from the remaining pixels (Fig 2). This procedure ensured that sample rows of terrorist attacks that resulted in casualties occupied only half of the entire sample. Finally, we can obtain 15970 sample data. To train and test the performance of machine learning models, three-quarters of the sample data were randomly selected as training data, and the remaining data were used as the test data, which were almost evenly distributed within the whole world.

### Machine-learning algorithms

We used version 3.30 of the 64-bit version of R, an open-source language and software for statistical computing[20], to build models, tune parameters and perform accuracy assessments. Three relatively popular and robust machine learning algorithms (NNET, SVM and RF) were adopted in the present study. The “caret” package[21] and built-in functions were used to train each model and tune their associated parameters within a single consistent environment.

For NNET, we use the backward propagation neural network (BPNN) algorithm, which has a powerful synaptic modification rule that will allow an arbitrarily connected neural network to develop an internal structure that is appropriate for a particular task domain[22]. Studies of BPNN suggest that increasing the number of hidden neurons improves classification accuracy[23, 24] and a large decay rate results in non-convergence[22]. Thus, the value of decay and the number of hidden units of the hidden layer were tuned when running a backward propagation neural network with sigmoid activation functions in the hidden and output layers.

For SVM based simulation, a radial basis function (RBF) kernel was considered for use in this research. Previous studies of the SVM algorithm had emphasized that two parameters (cost, sigma) used in the RBF kernel have an impact on classification accuracy[25]. A large cost means assigning a higher penalty to errors[26], whereas increasing sigma affects the shape of the separating hyperplane[27]. Therefore, we needed to fine-tune the cost and sigma parameters when performing simulation with the RBF kernel.

For simulation built with the RF model, the default number of trees (500) was used because values larger than 500 were unable to significantly improve the performance of the RF algorithm[28]. The *m*_*try*_ parameter controls the number of variables randomly sampled as candidates at each split and is known to have an effect on the overall classification accuracy[29]. For the binary partitioning, a small number of randomly selected variables are available, such as the value of the *m*_*try*_ parameter, which equals the square root of the number of variables within a dataset[30]. In the present study, only the *m*_*try*_ parameter was tuned when running the RF algorithm.

## Results

**Fig 3:**
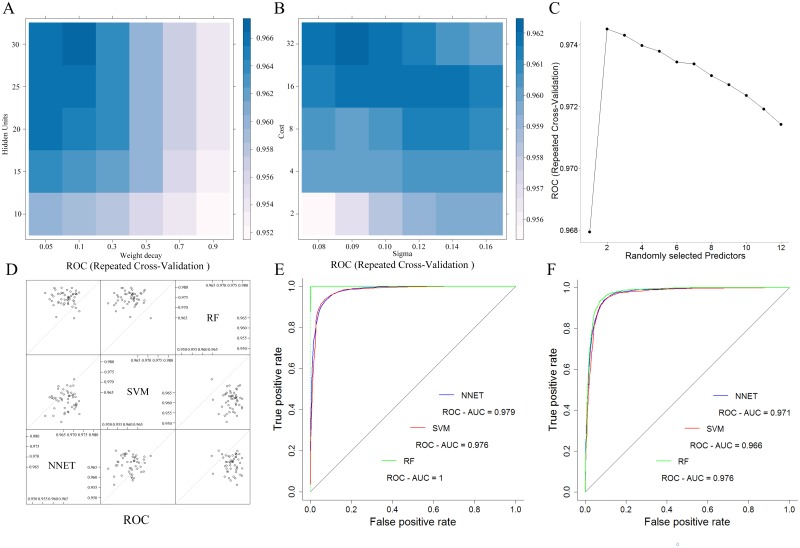
Selecting the optimal models using five repetitions of a 10-fold cross-validation. (A) Tuning the parameters of NNET. (B) Tuning the parameter of SVM. (C) Tuning the parameter of RF. (D) Comparing the performance of multiple models based on the same versions of the training data during the cross-validation process. (E) Receiver operating characteristic (ROC) curves of NNET, SVM and RF applied to training samples. (F) ROC curves of NNET, SVM and RF applied to validation samples.

Before using machine learning models to simulate the risk of terrorist attacks, we need to tune some parameters. The area under the curve (AUC) is used as a metric for measuring the performance of models. Larger AUC values indicate higher accuracy. Based on this criteria, we selected models with optimized tuning parameter values. For the NNET model, the highest AUC value (0.966) was obtained with 30 hidden units and a decay value of 0.1 (Fig 3A). For the simulation using SVM model, a total of 5 values for the cost parameter (2, 4, 8, 16 and 32) and 6 values for the sigma parameter (0.08, 0.09, 0.1, 0.12, 0.14 and 0.16) were examined. Based on the highest AUC obtained (0.962), a cost value of 32 was selected for the SVM model, whereas the value for the sigma parameter was set at 0.09 (Fig 3B). A total of 12 *m*_*try*_ parameter values (1, 2, 3, 4, 5, 6, 7, 8, 9, 10, 11 and 12) were examined for the simulation based on RF model. The highest AUC value (0.974) was obtained with a *m*_*try*_ value of 2 for the RF model (Fig 3C). Visualizing the resampling distributions, the RF model has better performance than the NNET and SVM models during the cross validation process on training samples. Moreover, the optimized models achieved an AUC value of over 0.95, which proved that there were some patterns between terrorist attacks and 12 factors (Fig 3D).

Based on a comparison between machine learning models applied to training datasets, we find that the RF model obtained the highest AUC (1) value, followed by the NNET (0.980) and SVM (0.976) models (Fig 3E). The same general trend was observed for machine learning models applied to validation datasets; the RF model had the highest AUC (0.976) value, followed by the NNET (0.971) and SVM (0.966) models (Fig 3F). In addition, the McNemar[31] test was used to assess whether a statistically significant difference existed between different machine learning models when using the validation samples. The McNemar test shows that the difference between the machine learning models based on the validation data was not significantly different (p>0.05).

**Fig 4:**
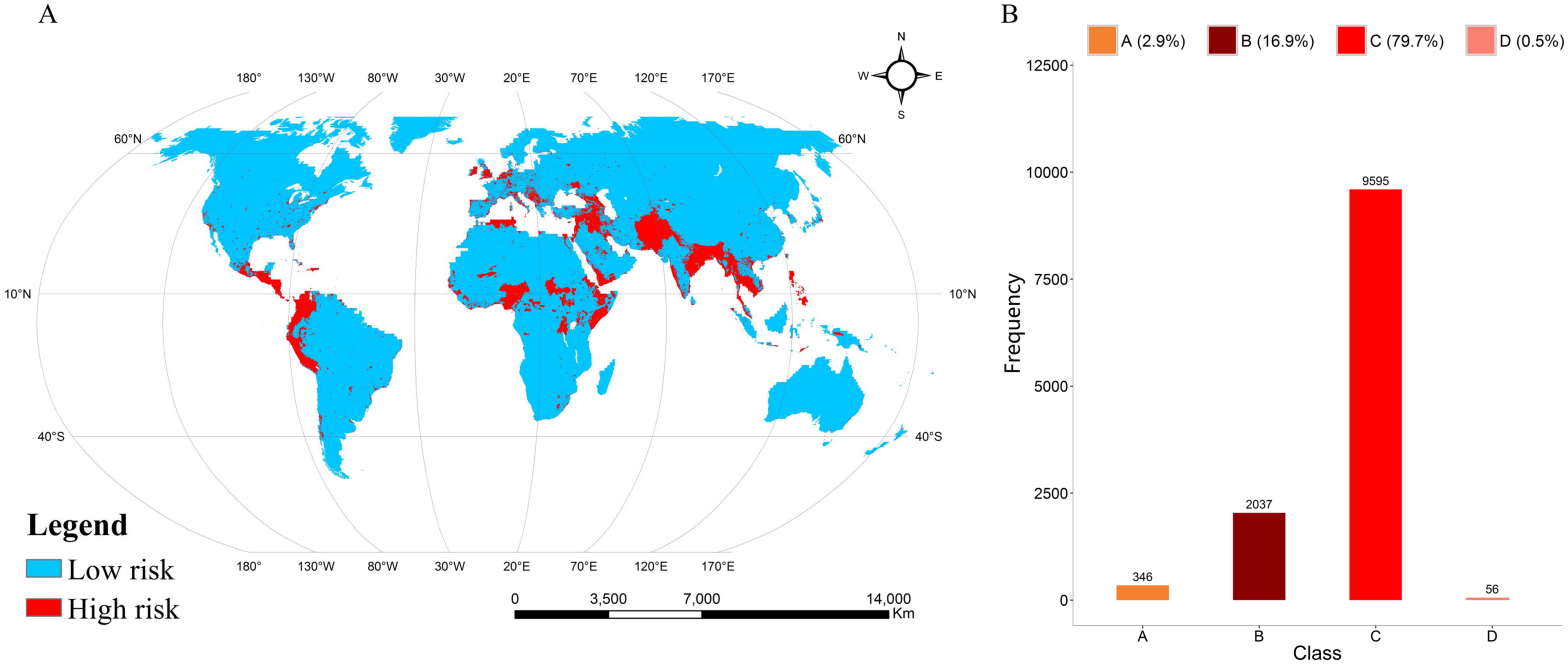
Simulation and verification. (A) The risk of terrorist attacks predicted by the RF-based model. (B) Verification of the prediction accuracy of the RF model using terrorist attacks data in 2015. Terrorism event locations that have no history of terrorist attacks in the past and lie in the low risk region of the prediction map belong to A. Terrorism event locations that have no history of terrorist attacks in the past and lie in the high risk region of the prediction map belong to B. Terrorism event locations that have a history of terrorist attacks and lie in the high risk region of the prediction map belong to C. Terrorism event locations that have a history of terrorist attacks and lie in the low risk region of the prediction map belong to D.

According to the results of the comparative analysis, we utilized the RF model, which obtained the best performance, to predict the risk of terrorist attacks in all regions worldwide. The results demonstrated that the high risk zones of terrorist attacks are mainly concentrated in South Asia, the Middle East, Central Africa, Northern Africa, Northwestern South America, Southern North American, Southern Europe and Western Europe (Fig 4A).

We divide the validation results derived from terrorism events that happened in 2015 into 4 categories (A, B, C, D) and emphasize that some terrorist attacks that happened in units that are missing some factors’ information are excluded in this step. Generally, 11,632 terrorist attacks happened in high risk regions, and the prediction accuracy of the RF model was 96.6%. More importantly, the RF model successfully predicted 2,037 terrorism event locations where a terrorist attack did not occur between 1970 and 2014 but occurred in 2015 (Fig 4B). In addition to this, the optimal model performed fairly well in predicting the places where terror events might occur in 2013 and 2014, with success rates of 96.0% and 94.7%, respectively.

**Fig 5:**
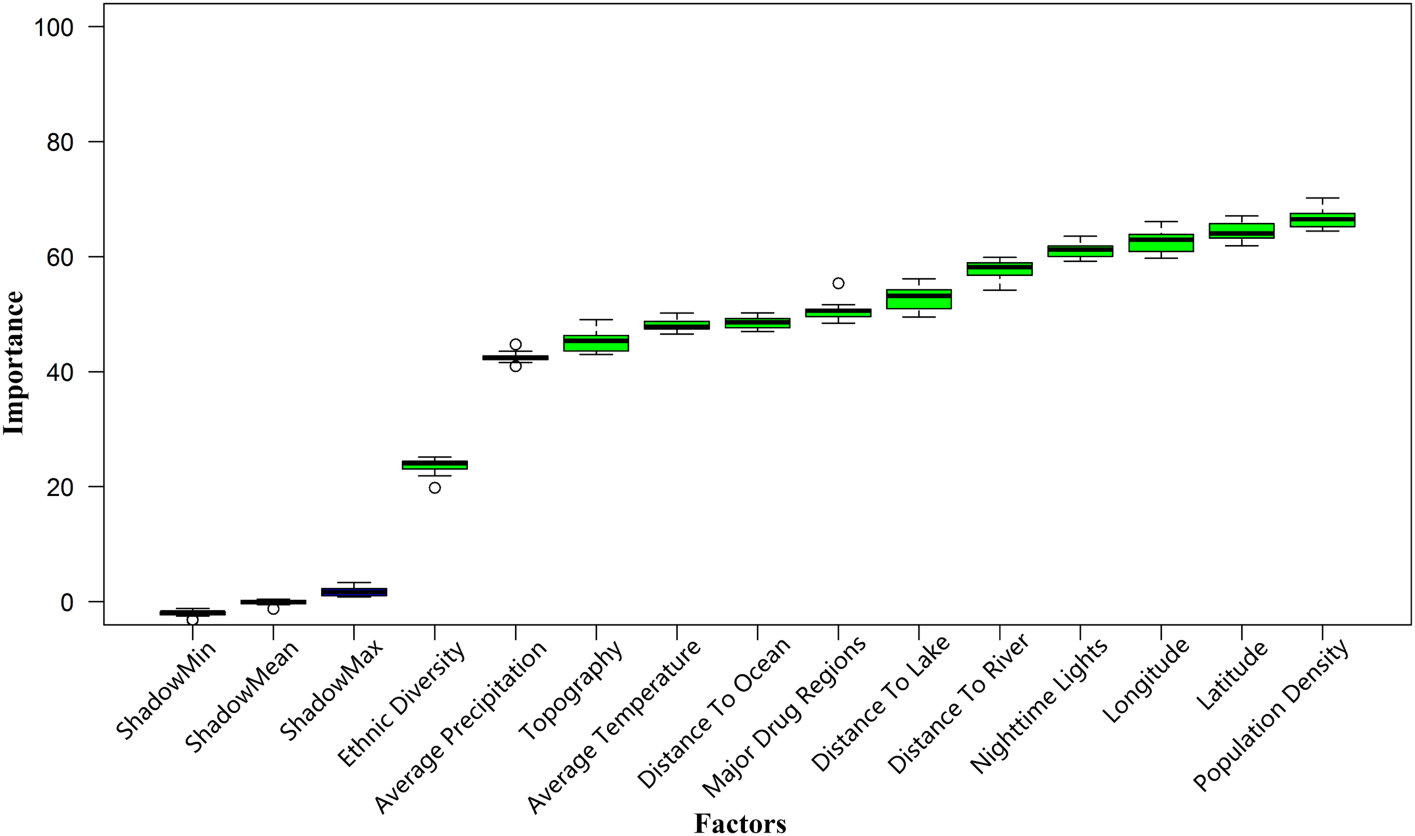
Measuring the discriminatory power of each factors.

Furthermore, we were also interested in understanding the mechanism between the 12 factors and terrorist attacks. Thus, we tried to statistically analyze the discriminatory power of each factors. The measurement was performed using the “Boruta” package[32], the default value (0.01) of confidence level was used and the maximal number of importance source runs was set to 100. The Fig 5 shows that the above geographical, natural and social factors are essential for simulation the risk of terrorist attacks in global regions. Additionally, the result suggests that the population density, nighttime lights and the location (latitude, Longitude) of the units play more important roles than the remaining factors in distinguishing whether a pixel belongs to a high risk unit, which is similar to that derived from F-score method. The population density factor has the highest mean importance (66.49), followed by latitude (64.45), longitude (62.50), nighttime lights (61.13), distance to a major navigable river (58.88), distance to a major navigable lake (53.28) and major drug region (52.86). Given the mean importance of major drug region, it may be reasonable and explainable by the reference 8, which writes that terrorist groups tax the drug trade as a source of funds.

## Conclusions

In this work, we demonstrate a novel strategy based on large amounts of data that uses machine learning models that are relatively typical and robust, though not the most advanced, to simulate the risk of terrorist attacks in all regions worldwide, which proved to be reasonable. Notably, the successful prediction of the risk of terrorist attacks using machine learning models was critically dependent on large volumes of high-dimensional data, including geographical, natural and social factors, which are quantized into space using dataset preprocessing (see Methods). Additionally, we recognize the contributions of other researchers made to the data source (Table 1). These data may not be the most representative in simulation risks of terrorist attacks worldwide, but it can be downloaded for free and quantified into the space. Moreover, the importance of each factors is summarized based on Boruta algorithm, the results of which are data-driven and the specific reason for those need further study.

Unfortunately, some terror events happened during this research, such as the Nice attack, the Brussels bomb attacks, the Orlando nightclub attack, the Wuerzburg train attack, and the Munich shootings, which caused a large number of casualties. To further verify the correctness of the prediction, we tested the locations of these terrorist incidents against the prediction results, which shows that the above terrorist attacks happened in high risk areas. Moreover, no significant difference was found among machine learning models (NNET, SVM and RF) based on the validation samples.

The next step will focus on the finer spatial and temporal scales to analyze the terror events, such as the distribution of terrorist attacks in different regions and the characteristics of terror attacks between the different years. Additionally, we will make better use of information from the GTD instead of using only the position and the number of casualties of each event when using machine learning models with rich data, which might reveal more key principles hidden in data.
